# Epilepsy and Neurodevelopmental Outcomes in Children With Etiologically Diagnosed Central Nervous System Infections: A Retrospective Cohort Study

**DOI:** 10.3389/fneur.2019.00528

**Published:** 2019-05-15

**Authors:** Chien-Heng Lin, Wei-De Lin, I-Ching Chou, Inn-Chi Lee, Syuan-Yu Hong

**Affiliations:** ^1^Division of Pediatrics Pulmonology, China Medical University, Children's Hospital, Taichung, Taiwan; ^2^Department of Biomedical Imaging and Radiological Science, China Medical University, Taichung, Taiwan; ^3^Department of Medical Research, China Medical University Hospital, Taichung, Taiwan; ^4^Division of Pediatrics Neurology, China Medical University, Children's Hospital, Taichung, Taiwan; ^5^College of Chinese Medicine, Graduate Institute of Integrated Medicine, China Medical University, Taichung, Taiwan; ^6^Department of Pediatrics, Chung Shan Medical University Hospital, Taichung, Taiwan; ^7^School of Medicine, Institute of Medicine, Chung Shan Medical University, Taichung, Taiwan

**Keywords:** epilepsy, neurodevelopmental outcomes, central nervous system infections, brain infections, children

## Abstract

**Background:** Central nervous system (CNS) infection in childhood can lead to neurological sequelae, including epilepsy, and neurodevelopmental disorders, such as attention-deficit/hyperactivity disorder (ADHD) and autism spectrum disorder (ASD). This study investigated the association of etiologically diagnosed childhood brain infections with the subsequent risks of epilepsy and neurodevelopmental disorders.

**Objectives:** We retrospectively analyzed the data of children aged <18 years who had definite brain infections with positive cerebrospinal fluid cultures from January 1, 2005, to December 31, 2017. These patients were followed to evaluate the risks of epilepsy and neurodevelopmental disease (ADHD and ASD) after brain infections (group 1) in comparison with the risks in those without brain infections (group 2).

**Results:** A total of 145 patients with an average age of 41.2 months were included in group 1. *Enterovirus* accounted for the majority of infections, followed by group B *Streptococcus, S. pneumoniae*, and herpes simplex virus. A total of 292 patients with an average age of 44.8 months were included in group 2. The 12-year risk of epilepsy in group 1 was 10.7 (95% confidence interval [CI], 2.30–49; *p* < 0.01). Compared with group 2 (reference), the risk of ASD in the age interval of 2–5 years in group 1 was 21.3 (95% CI, 1.33–341.4; *p* = 0.03). The incidence of ADHD in group 1 was not significantly higher than that in group 2.

**Conclusions:** This study identified the common etiological causes of brain infections in Taiwanese children. The highest-risk neurodevelopmental sequelae associated with brain infections was epilepsy. Children who had a diagnosis of brain infection (specially Enterovirus) should be followed since they are at greater risk of developing epilepsy and ASD.

## Introduction

Central nervous system (CNS) infections in childhood can be devastating and may lead to neurological sequelae or death. However, different pathogens imply different neurological prognoses. A greater understanding of causative agents may lead to greater opportunities to predict associated neurological outcomes of children later in life (1). The risk of unprovoked seizures after encephalitis and meningitis has been explored in a previous study (2). Although seizures occur frequently in children with acute bacterial meningitis, only those with permanent neurologic deficits after meningitis are at a high risk of epilepsy (3, 4). Seizures also occur in children with acute viral encephalitis and increase the risk of later unprovoked seizures and epilepsy (5).

Neurodevelopmental disorders (NDDs) are a group of conditions that interfere with CNS development at an early age. Developmental brain dysfunction causes later neuropsychiatric problems that persist into adulthood. There are various types of NDDs; common NDDs in children are attention-deficit/hyperactivity disorder (ADHD), autism spectrum disorder (ASD), learning disabilities, and intellectual disability (6).

The pathogenesis of epilepsy and NDDs in children has not been completely understood. It is generally known that they are caused by several factors, including genetic factors, neurobiological factors, environmental and perinatal factors, parental age, and maternal medication use during pregnancy (7). Among them, CNS infections play a pivotal role as environmental and perinatal factors (8).

The impact of CNS infection in childhood is considerably different from that in adulthood. Children acquire CNS infections through various routes and at different time points. Maternal infections during pregnancy, regardless of viral, bacterial, or parasitic illnesses, have been shown to be associated with fetal brain malformations, including intracranial calcifications, cortical atrophy, microcephaly, and hydrocephalus. Simultaneously, the inflammatory pathways caused by infections lead to the release of various inflammatory cytokines and morphological changes consistent with an infectious intrauterine environment or umbilical cord, causing the immature CNS to be at risk (9). When newborn infants move through an infected birth canal, they are susceptible to encephalitis, especially that caused by HSV and GBS. Neurological dysfunction associated with brain infections during CNS development often exceeds cellular damage directly attributable to virus replication and thus results in permanent neurological deficits (10). In addition, neurologic symptoms of CNS infection in young children can be subtle and indolent and can easily be missed owing to the lack of classic adult symptoms (11). When CNS infection diagnosis is delayed in an infant or child, the consequences can be harmful. Some evidence suggests that brain infections in infancy or childhood compromises early CNS development and might increase the risk of epilepsy and other NDDs later in life (12–15).

This study explored the neurodevelopmental outcomes of childhood brain infections with different microbiological etiologies, with the aim of developing strategies of prevention, early diagnosis, and targeted treatments for epilepsy and NDDs after CNS infections in children.

## Materials and Methods

In this retrospective cohort study, we analyzed patients <18 years with clinically suspected CNS infections and collected the data of them who had definite brain infections with positive cerebrospinal fluid (CSF) cultures between January 1, 2005, and December 31, 2017. The preliminary inclusion protocols based upon the following criteria (16):

Altered mental status lasting ≥24 h with no alternative cause identified, plus ≥2 of the following for a “possible” diagnosis or ≥3 of the following for a “probable” diagnosis:

1. Documented fever ≥38°C (100.4°F) within 72 h (before or after) presentation.
2. Generalized or partial seizures not fully attributable to preexisting seizure disorder.
3. New onset focal neurologic findings.
4. CSF WBC count ≥5 cells/microL.
5. Abnormality of brain parenchyma on neuroimaging suggestive of encephalitis that is new or appears to have acute onset.
6. Abnormality on EEG that is consistent with encephalitis and not attributable to another cause.

A comprehensive medical record review was strictly enforced to exclude the children who had either epilepsy, neurologic, metabolic, autoimmune, or any other congenital disorders before the onset of CNS infection. Individuals who had more than one episode of CNS infection were also excluded from the final analysis. The last subject was enrolled in December 2017. All patients included in the study (study and controls) had a follow-up period began from baseline (the time point they were included in the study) until the end of follow-up (October 30, 2018); or death. We conducted the follow-up measures through medical records, telephone or e-mail access to the families quarterly. Once a suspected NDD was noticed, we contacted the children returning to our Pediatric neurology clinic for a comprehensive assessment.

To focus on bacteria and certain viruses that can be cultured from CSF, we sent patient's CSF for Gram stain, bacterial culture and viral studies (viral culture, polymerase chain reaction).

A positive CSF microbial etiology was defined as one yielding pathogens from CSF through multiplex polymerase chain reaction testing and viral and bacterial culture (17). To determine the key infectious etiology, patients with more than one pathogen isolated from CSF underwent the following to obtain more clinical information: the culture of other sites or CSF and serum antibody testing. After the screening process, the final study population comprised 145 subjects. Among the study subjects, we also looked into the issues during their admission which may be related to long-term neurological sequelae: With or without ventilator use; 1st day Pediatric Glasgow Coma Scale, seizures pattern (if any); type of Brain infections (by analyzing their neuroimaging or by clinical judgment of individual physicians), with a view to see the association between various brain infections (meningitis, encephalitis, abscess, etc.) and clinical risk factors at admission with subsequent NDDs.

**Figure 1:**
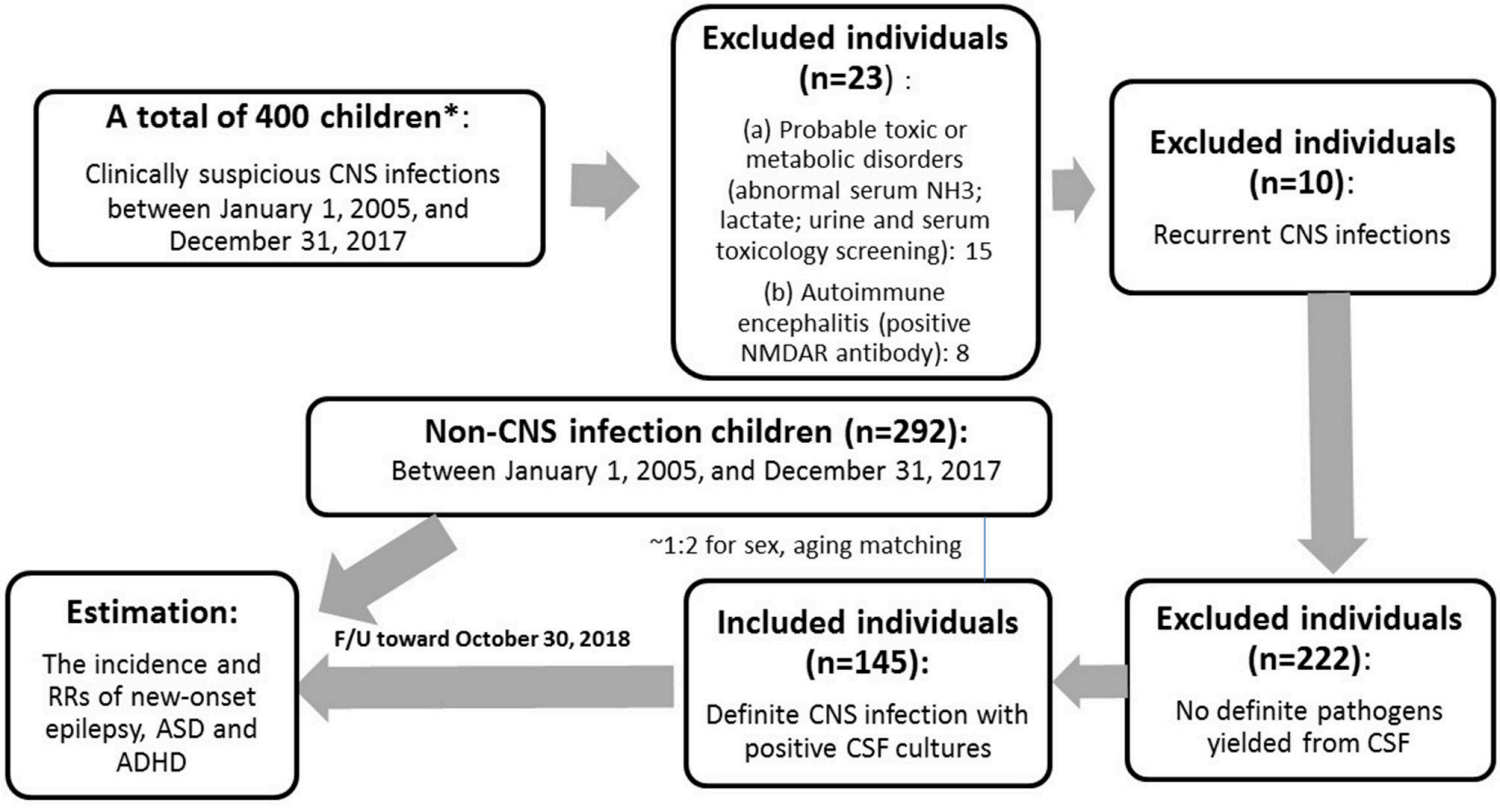
Flowchart of the study. *The patients who were screened from the medical records and contacted by the our case managers.

Instruments used in the assessment of children and adolescents with suspected intellectual disability (ID) were: Bayley Scales of Infant and Toddler Development, 3rd Edition (for young children <2 years), Wechsler Preschool and Primary Scale of Intelligence (for children ages 2 years 6 months to 7 years 7 months) and Wechsler Intelligence Scales for Children, 5th Edition (WISC-V) (for children in school age and beyond). Epilepsy (our outcome of interest) was defined as 2 unprovoked seizures more than 24 h apart whose diagnosis was made by a Pediatric neurologist. Patients who met the relevant diagnostic criteria in the Fourth and Fifth Editions of *Diagnostic and Statistical Manual of Mental Disorders* were diagnosed with ASD and ADHD, and their diagnosis was made by a Pediatric psychiatrist or Pediatric neurologist in the inpatient or outpatient setting of China Medical University Children's Hospital between January 1, 2005, and December 31, 2017. We selected 292 children with unspecified infections (acute gastroenteritis, acute tonsillitis, urinary tract infection, and others) between January 1, 2005, and December 31, 2017, whose positive culture results were yielded from throat, urine, stool, or other body discharges but not from blood or CSF as a matched control group in terms of age and mean follow-up years. The control group was derived from the outpatient clinic and had been confirmed to have fully recovered from their mild illness during follow-up (Figure 1).

Gender, age of infection onset, and associated pathogens constituted confounding factors. The study population was divided into 4 groups based on the age of infection onset: ages 0–2 years (infants and toddlers), ages 2–5 years (preschoolers), ages 5–10 years (young children), and ages >10 years (young teens and teenagers) (Table 1). The study protocol was approved by the Ethics Review Board of the China Medical University Ethics Committee. Categorical variables between groups were analyzed using χ^2^ tests. Moreover, we calculated the incidence density rate of epilepsy, ADHD, and ASD in the control group and the brain infections group (which was subdivided into different age intervals and subdivided by pathogens). Using risk regression model, we estimated of the risk ratios (RRs) and 95% confidence intervals (CIs) of epilepsy, ADHD, and ASD in the brain infections group relative to the control group. All statistical analyses were performed using PASW Statistics version 18.0 software (SPSS Inc., Chicago, IL, USA). In addition, for all executed statistical analyses, we deemed 2-tailed *p* < 0.05 to indicate statistically significant results.

**Table 1:** Comparison of demographics between brain infections and controls.

	**Group**		***p***
	**Brain infections** **(*N* = 145, %)**	**Controls** **(*N* = 292, %)**	
Mean follow-up years to epilepsy (SD)^*^	7.7 (3.6)	7.9 (3.5)	>0.99
Mean follow-up years to ADHD (SD)^*^	7.6 (3.8)	7.8 (3.6)	>0.99
Mean follow-up years to ASD(SD)^*^	7.7(3.8)	7.9 (3.7)	>0.99
Gender			0.28
Male	98 (67)	182 (62)	
Female	47 (33)	110 (38)	
Infection mean age (months) (SD)	41.2(60.70)	44.8 (41.24)	0.46
Stratified by age (yr)			>0.99
0–2	92 (63.4)	184 (63)	
2–5	15 (10.3)	31 (10)	
5–10	16 (11.0)	33 (11)	
>10	22 (15.1)	44 (15)	
Associated pathogens			N/A
Enterovirus	78 (53.7)	_	
Herpes simplex virus	8 (5.5)	_	
Group B streptococcus	28 (19.3)	_	
S. pneumoniae	10 (6.8)	_	
Others	21 (14.4)	_	
Neurodevelopmental outcomes			<0.01
ID or CP	48 (33.1)	9 (3.0)	
Epilepsy	11 (7.5)	1 (0.3)	
ADHD	4 (2.7)	6 (2.0)	
ASD	1 (0.7)	0 (0)	
Epilepsy+ADHD	1 (0.7)	1 (0.3)	
ADHD+ASD	1 (0.7)	1 (0.3)	

* *t test*.

## Results

### Data Analysis

From January 1, 2005, to December 31, 2017, a total of 145 children who were diagnosed with brain infections with definite pathogens and 292 children with non- brain infections were enrolled into this study. Table 1 presents the participants' demographic factors. The participants' mean age was 3.43 years (standard deviation [SD], 5.05). The proportion of boys was higher than that of girls (67 vs. 33%). The mean (SD) follow-up years for participants with epilepsy, ADHD, and ASD were 7.7 (3.6), 7.6 (3.8), and 7.7 (3.8), respectively. The minimal follow-up duration was 11 months year since the last subject was included in December 2017 and the maximum follow-up duration was 13 years and 10 months since the first subject was included in January, 2005.

**Figure 2:**
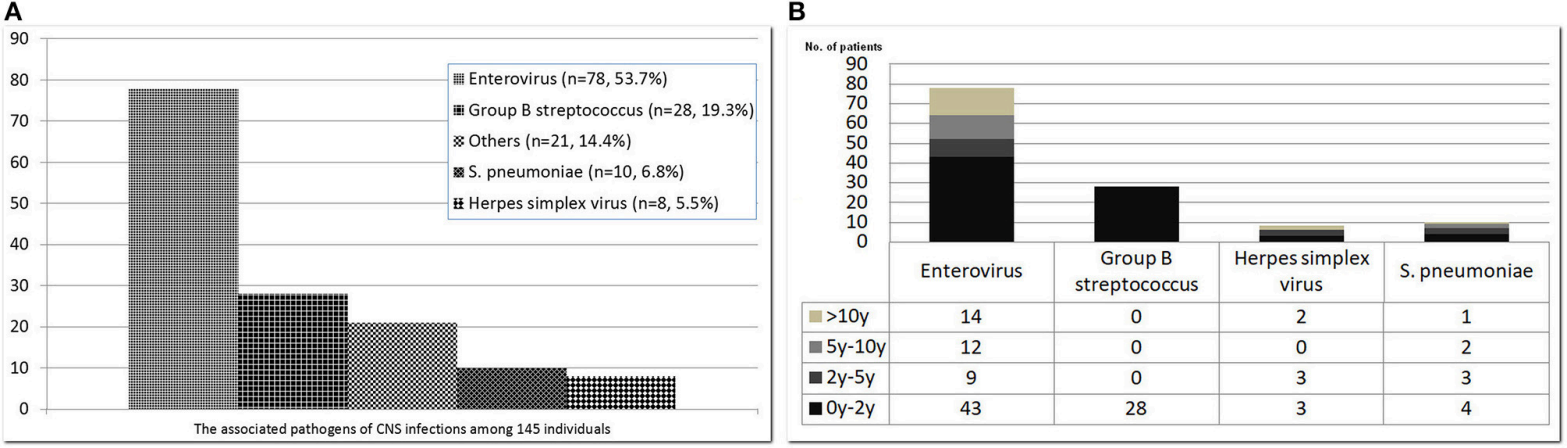
Common pathogens of central nervous system infections among 145 affected subjects **(A)** and their stratification by age **(B)**. The pathogenic organisms of “others” included Adenovirus (*n* = 2), HHV-6 (*n* = 2), HHV-7 (*n* = 1), Parvovirus (*n* = 1), Varicella-zoster virus (n = 1), Citrobacter koseri (n = 1), Enterobacter hormaechei (n = 1), Enterococcus casseliflavus (n = 1), Escherichia coli (*n* = 2), Haemophilus influenzae (*n* = 2), Neisseria meningitidis (*n* = 1), Listeria monocytogenes (*n* = 1), Mycobacteria tuberculosis complex (*n* = 1), Staphylococcus aureus (*n* = 2), Streptococcus constellatus (*n* = 2).

**Figure 3:**
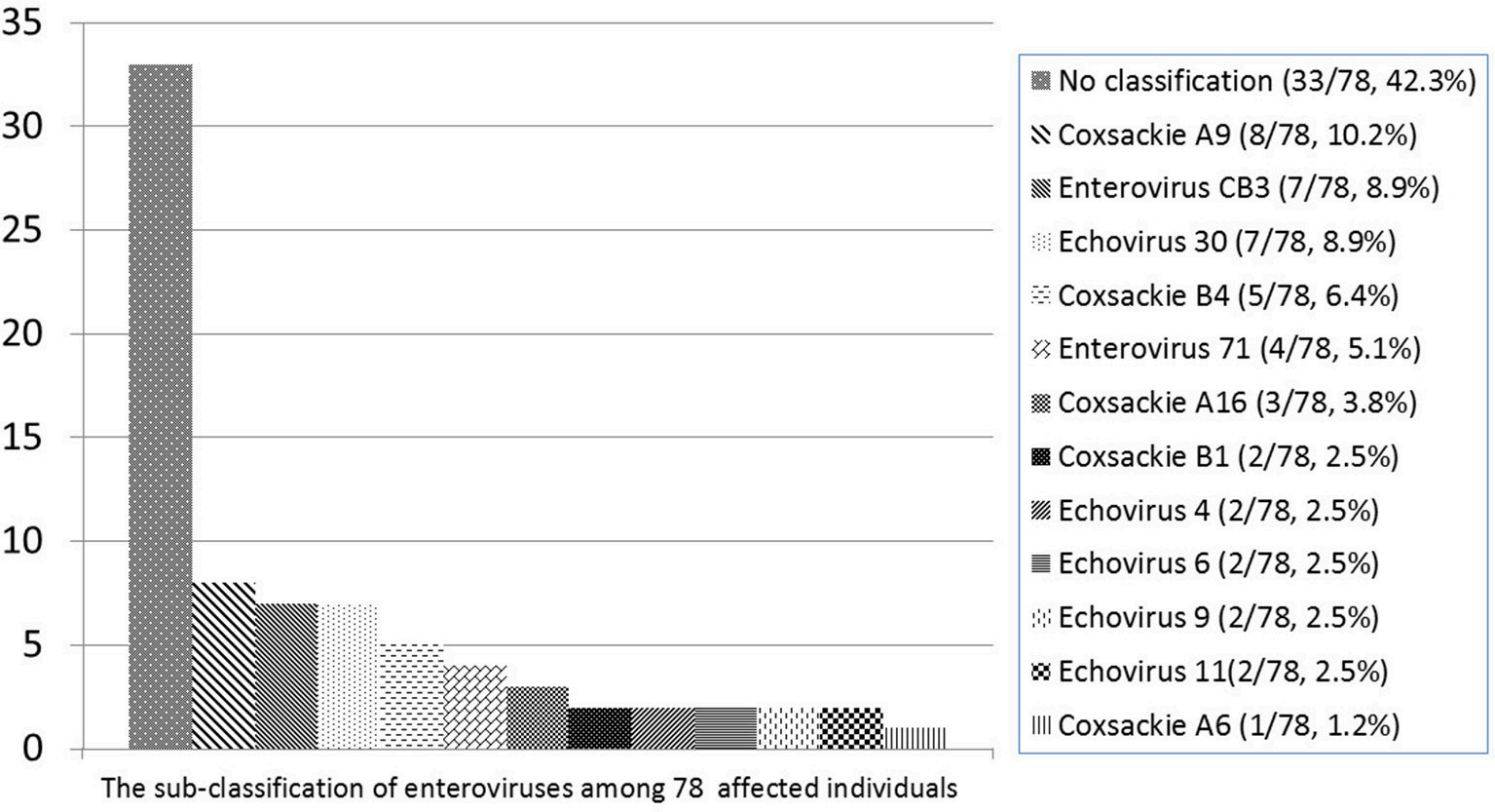
Subclassification of enteroviruses among 78 affected individuals.

The common pathogens of brain infections in this study were enteroviruses (*n* = 78, 53.7%), herpes simplex virus (HSV) (*n* = 8, 5.5%), group B *Streptococcus* (GBS) (*n* = 28, 19.3%), *Streptococcus pneumoniae* (*n* = 10, 6.8%), and others (*n* = 21, 14.4%) —namely, Adenovirus (*n* = 2), HHV-6 (*n* = 2), HHV-7 (*n* = 1), Parvovirus (*n* = 1), Varicella-zoster virus (*n* = 1), Citrobacter koseri (*n* = 1), Enterobacter hormaechei (*n* = 1), Enterococcus casseliflavus (*n* = 1), Escherichia coli (*n* = 2), Haemophilus influenzae (*n* = 2), Neisseria meningitidis (*n* = 1), Listeria monocytogenes (*n* = 1), Mycobacteria tuberculosis complex (*n* = 1), Staphylococcus aureus (*n* = 2), Streptococcus constellatus (*n* = 2) (Figure 2). The subclassification of enteroviruses is shown in Figure 3. The incidence rates of epilepsy and neurodevelopmental outcomes for brain infections vs. controls populations were as follows: 7.5 vs. 0.3 (epilepsy), 2.7 vs. 2.0 (ADHD), 0.7 vs. 0 (ASD), 0.7 vs. 0.3 (epilepsy + ADHD), and 0.7 vs. 0.3 (ADHD + ASD). We did not find children with both epilepsy and ASD in the brain infections group and the control group.

### Neurodevelopmental Outcomes

**Figure 4:**
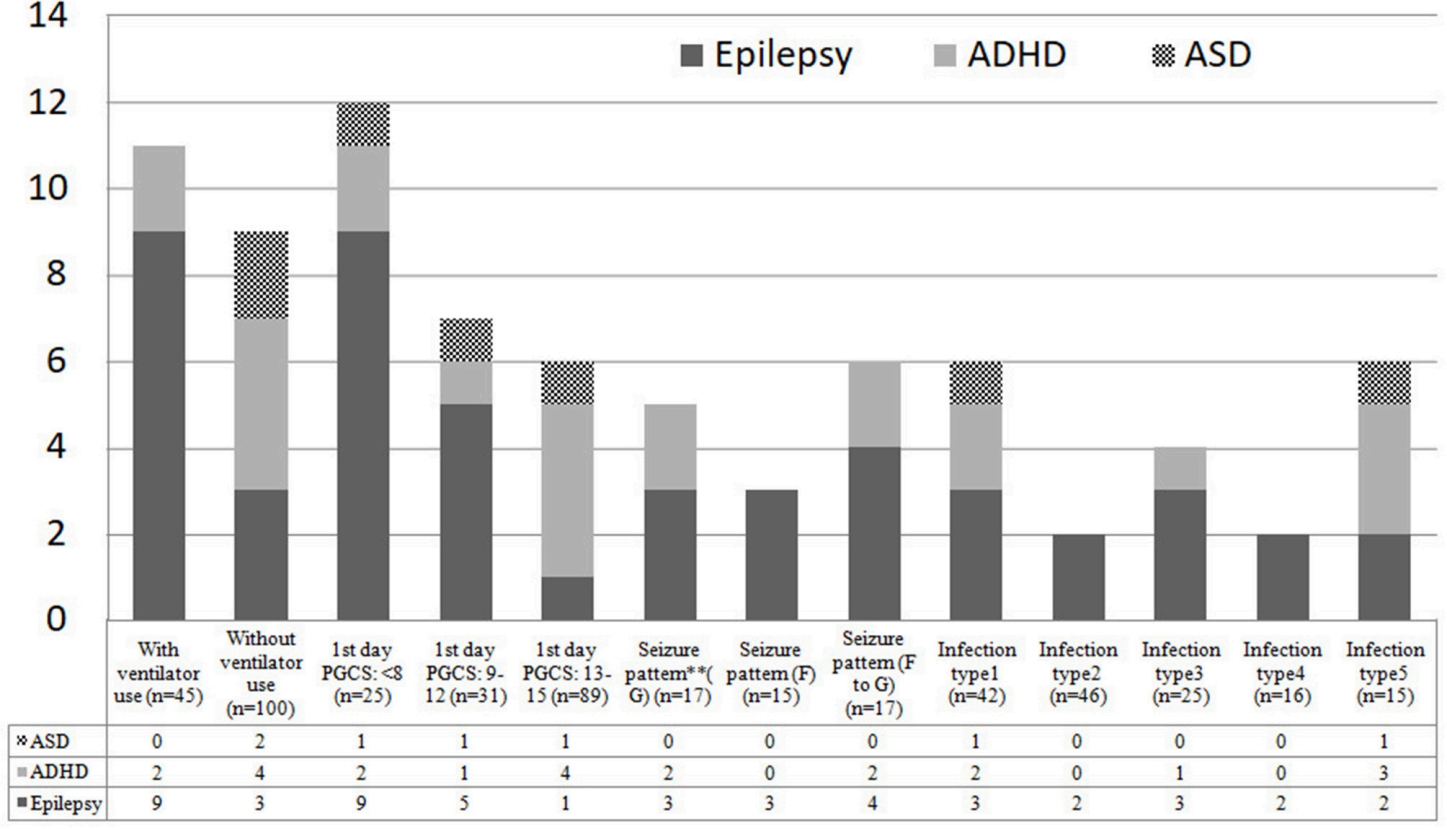
Possible risk factors during admission for subsequent NDDs among 145 study children. PGCS, Pediatric Glasgow Coma Scale; F, focal; G, generalized; F to G, focal seizures evolving to secondarily generalized seizures; Infection type 1: meningitis; Infection type 2: Encephalitis; Infection type 3: meningoencephalitis; Infection type 4: Encephalomyelitis; Infection type 5: other brain infections types. ** Seizure patterns were classified into focal, generalized, focal to generalized, according to their clinical features of seizures and electroencephalographic data.

The possible risk factors during hospitalization associated with epilepsy and NDDs were illustrated in Figure 4. Intellectual disability and/or cerebral palsy account for the majority of long-term neurological sequelae throughout all possible prognostic markers. The incidence density rates of epilepsy were compared between the brain infections group and the control in analyses stratified by several variables, including age of CNS infection onset and associated pathogens. The overall risk of epilepsy in the brain infections group was higher than that in the control group (RR, 10.7; 95% CI, 2.30–49.8; *p* = 0.002). Moreover, in the stratified analysis, the risk of epilepsy in the brain infections group was higher than in the control group for all age ranges (except for children >10 years old) and for all different pathogens tested (Table 2).

**Table 2:** Incidence rates and relative risks of epilepsy for the controls compared with brain infections cohort.

	**Event**	**IR (%)**	**RR (95% CI)**
Controls	2	0.68	Reference
All brain infections group	12	8.27	10.7 (2.30–49.8)^**^
Brain infections at (yr)			
0–2	9	6.20	12.3 (2.56–59.69)^**^
5–10	1	0.68	16.20 (2.21–118.38)^**^
>10	2	1.37	7.52 (0.67–84.30)
Brain infections with different pathogens			
Enterovirus	4	2.75	6.04 (1.05–34.55)^*^
Herpes simplex virus	1	0.68	22.3 (2.00–249.93)^**^
Group B streptococcus	2	1.37	9.90 (1.35–72.30)^*^
S. pneumoniae	1	0.68	14.70 (1.50–68.40)^*^
Others	4	2.75	6.04 (1.05–34.55)^*^

*IR, incidence rate; RR, risk ratio; yr, year; -, No event; N/A, not available*.

* *P < 0.05*.

** *P < 0.01*.

#### Incidence Rates and Relative Risks of ADHD

Overall, no significant difference in the incidence rates and relatives risks of ADHD were noted between the brain infections group and the control group (RR, 1.51; 95% CI, 0.53–4.27; *p* = 0.56) as well as in stratified analyses (Table 3).

**Table 3:** Incidence rates and relative risks of ADHD for the controls compared with brain infections cohort.

	**Event**	**IR (%)**	**RR (95% CI)**	***p***
Controls	8	2.73	Reference	
All brain infections group	6	4.12	1.51 (0.53–4.27)	0.56
Brain infections at (yr)				
0–2	3	2.06	0.79 (0.19–3.24)	0.99
2–5	2	1.37	5.93 (0.90–38.9)	0.99
5–10	1	0.68	3.93 (0.26–59.5)	0.36
Brain infections with different pathogens				
Enterovirus	5	3.44	2.34 (0.78-6.95)	0.16
Others	1	0.68	1.52(0.198-11.6)	0.51

*IR, incidence rate; RR, risk ratio; yr, year; -, No event; N/A: not available*.

#### Incidence Rates and Relative Risks of ASD

Overall, no significant difference in the incidence rates and relative risks of ASD was noted between the brain infections group and the control group (RR, 4.7; 95% CI, 0.42–51.7; *p* = 0.20). However, notably, the incidence rates and relative risks of ASD were significantly higher in the children in the age interval of 2–5 years (RR, 21.3; 95% CI, 1.33–341.4; *p* = 0.03) (Table 4).

**Table 4:** Incidence rates and relative risks of ASD for the controls compared with brain infections cohort.

	**Event**	**IR (%)**	**RR (95% CI)**	***p***
Controls	1	0.34	Reference	
All brain infections group	2	1.37	4.7 (0.42–51.7)	0.20
Brain infections at (yr)				
0–2	1	0.68	3.77 (0.23–60.3)	0.34
2–5	1	0.68	21.3 (1.33–341.4)^*^	0.03
Brain infections with different pathogens				
Enterovirus	2	1.37	8.06 (0.73–88.9)	0.08

*IR, incidence rate; RR, risk ratio; yr, year; -, No event; N/A, not available*.

* *P < 0.05*.

## Discussion

Although the identification of a pathogen known to cause encephalitis or meningitis confirms the diagnosis, in cases with negative CSF cultures, the diagnosis of viral encephalitis is made mostly based on clinical judgment when altered mental status and clinical presentation of viral encephalitis are suspected and abnormal CSF, electroencephalographic, or neuroimaging findings are obtained. Similarly, remarkable CSF results usually are sufficient to support the diagnosis of bacterial meningitis (16, 18).

However, identifying the causative pathogen in brain infections in children is crucial not only for treatment but also for neurodevelopmental prediction (19, 20). We focused on only three pathogenic organisms and found that *Enterovirus* accounted for the majority of brain infections, followed by GBS, *S*. *pneumoniae*, and HSV. Among the NDD studied the highest risk neurodevelopmental sequel was epilepsy among all pathogen classifications and most age intervals. Only children with enteroviral encephalitis during the preschool age interval had a higher risk of subsequent ASD.

Up to now, a small number of studies have explored the association between specific neurotropic pathogen infections during childhood and NDDs (15, 21–27). Among these studies, a study enrolled 86 children with viral culture–confirmed *Enterovirus 71* (EV71) infection (15). That study found that those who had been infected with EV71 had a higher risk of developing ADHD-related symptoms. However, the study did not investigate the association of ADHD with other types of *Enterovirus*. According to Huang et al. (26), different type of Enterovirus infection might be responsible for different neurodevelopmental and behavior disorders (EV71 is associated with ADHD, delayed neurodevelopment, and reduced cognitive functioning; coxsackievirus increases the risk of schizophrenia) (1, 27). Our study did not obtain the same results, possibly because only 4 out of 78 participants were identified with EV71 and a comprehensive group of enteroviruses was studied.

Unprovoked seizures and epilepsy can be long-term sequelae of bacterial meningitis and their risk were relatively higher for Streptococcus pneumoniae (28). Misra et al. reviewed acute symptomatic and late unprovoked seizures (epilepsy) in different viral encephalitides, which found: epilepsy caused by Herpes simplex encephalitis is associated with poor prognosis; Japanese encephalitis is the most common one associated with acute symptomatic seizures in children; Late-onset Nipah virus encephalitis is more commonly having seizures than acute encephalitis be (5). Although maternal and prenatal infections have been recognized to be associated with an increased risk of ASD (13, 29), we propose that ASD may also be related to childhood enteroviral infection, especially during the preschool age interval. A potential explanation for this finding is that brain infections during these ages represent an exaggeration or abnormality of normal processes that occur during the completion of brain development, which may impede children's communication and social skills. Hence, we presume infection older than preschool age in which brain development has approached completeness, may be affected to a lesser extent (which could also be observed in epilepsy group, Table 2). However, we are not clear why those infections younger than preschool age did not have the statistical difference in ASD incidence. More research with larger number of samples and more rigorous experimental design are necessary to explore this issue. We also found that a small subset of patients had dual neurodevelopmental comorbidities (i.g. epilepsy+ASD or ADHD+ASD). Although the case number was too small to further analyze, recent studies has shown that NDDs have been considered to have high rates of both homotypic and heterotypic comorbidity, which needs to be taken into consideration in health service planning and treatment delivery (30, 31).

Other risk factors possibly associated with subsequent NDDs during admission were also identified in this study, including ventilator use, low Glasgow Coma Scale score in the first day, different infection types and patterns of seizure (Figure 4). Remarkably, the more critical the patients initially presented, the higher risk of subsequent NDDs they may have. Furthermore, additional research focusing on neuroimaging, genetic susceptibility, or proinflammatory cytokines and other environmental exposures may identify potential mechanisms underlying NDDs in children with different patterns of brain infections.

Our study had several limitations. First, as stated above, the causal role of pathogenic agents is sometimes difficult to determine from human CSF (e.g., influenza virus and cytomegalovirus), and these types of infection may have inevitably been lost under the present study design, which rendered us unable to obtain information on holistic neurological outcomes for all etiological brain infections. Second, several non-infectious causes, such as patient's immune status, neurogenetic factors, and structural or metabolic disorders, may be confounders for the development of epilepsy and NDDs in childhood and were not investigated in the present study. Third, because this was a retrospective longitudinal study that used patients' medical records obtained from a single medical center, patients who opted to receive medical care at another hospital or clinic were not included in this study, which may have affected the results. Fourth, RR for ASD (in children who had brain infection between 2 and 5 year age) was based on only one patient. In addition, only certain pathogens and NDDs were focused on. Therefore, more samples and diverse pathogens as well as types of NDDs should be included in the future research to support our finding.

In conclusion, our study identified common etiological causes of brain infections in Taiwanese children. Among them, *Enterovirus* accounted for the majority of infections, followed by GBS, *S*. *pneumoniae*, and HSV. In addition to ID, which is considered the most common neurological sequelae in brain infections, our study further shows that the highest-risk neurodevelopmental sequelae was epilepsy among all pathogen classifications and most age intervals. Children who had a diagnosis of brain infections (specially Enterovirus) should be followed since they are at greater risk of developing epilepsy and ASD. Only children with enteroviral encephalitis during the preschool age interval had a higher risk of ASD. Therefore, early assessment and identification of epilepsy and other NDDs facilitate early intervention and treatment among these children. Additional studies should examine the relationship between additional etiological sources and their possible associations with other NDDs, such as Tourette syndrome, communication disorders, and speech and language disorders.

## Ethics Statement

After a full description of the study, written informed consent of participation was obtained from the legal guardians. The study protocol was approved by the Ethics Review Board of the China Medical University ethics committee (Approval # CMUH108-REC1-023 and # CMUH107-REC2–017 and #CRS-106-027 and #CRS-106-031).

## Author Contributions

S-YH collected and analyzed the data and prepared the draft. C-HL and W-DL participated in the design of the study and wrote the manuscript. I-CL and I-CC compiled the statistics of this study and participated in the editing and revising the tables. All authors read and approved the final manuscript.

### Conflict of Interest Statement

The authors declare that the research was conducted in the absence of any commercial or financial relationships that could be construed as a potential conflict of interest.
